# Chair-Based Exercises for Frail Older People: A Systematic Review

**DOI:** 10.1155/2013/309506

**Published:** 2013-09-09

**Authors:** Kevin Anthony, Katie Robinson, Philippa Logan, Adam L. Gordon, Rowan H. Harwood, Tahir Masud

**Affiliations:** ^1^Falls and Bone Health Service, Nottingham CityCare Partnership, Newbrook House, 385 Alfreton Road, Nottingham NG7 5LR, UK; ^2^Division of Rehabilitation & Ageing, University of Nottingham, Queens Medical Centre, Nottingham NG7 2UH, UK; ^3^Health Care of Older People, Nottingham University Hospitals NHS Trust, Queens Medical Centre, Nottingham NG7 2UH, UK

## Abstract

*Introduction*. Frail older people are often unable to undertake high-intensity exercise programmes. Chair-based exercises (CBEs) are used as an alternative, for which health benefits are uncertain. *Objective*. To examine the effects of CBE programmes for frail older people through a systematic review of existing literature. *Method*. A systematic search was performed for CBE-controlled trials in frail populations aged ≥65 years published between 1990 and February 2011 in electronic databases. Quality was assessed using the Jadad method. *Results*. The search identified 164 references: with 42 duplicates removed, 122 reviewed, 116 excluded, and 6 analysed. 26 outcome measures were reported measuring 3 domains: mobility and function, cardiorespiratory fitness, mental health. All studies were of low methodological quality (Jadad score ≤2; possible range 0–5). Two studies showed no benefit, and four reported some evidence of benefit in all three domains. No harmful effects were reported; compliance was generally good. *Conclusion*. The quality of the evidence base for CBEs is low with inconclusive findings to clearly inform practice. A consensus is required on the definition and purpose of CBEs. Large well-designed randomised controlled trials to test the effectiveness of CBE are justified.

## 1. Introduction

Exercise has wide-ranging health benefits in older people [1, 2]. For community-dwelling populations there is clear evidence to support exercise in improving health and quality of life [3] with well-evidenced exercise programmes widely employed in clinical practice. These programmes have been shown to reduce the risk of falls with associated benefits on mortality, morbidity, and costs to health and social care [4, 5]. These programmes involve exercises performed whilst standing unaided and are often too challenging for older people with compromised balance and mobility. Pragmatic approaches have evolved that are tailored to meet the needs of frailer older people where exercise is performed primarily in the seated position-chair-based exercise (CBE). CBE programmes are often provided for older people with limited mobility in both residential care home and community settings [6]. This results from the belief that they lack the ability to engage or participate in higher-intensity progressively challenging standing exercises [6]. To ensure the quality and effectiveness of exercise provision for this specific population, practice should be guided by the best available evidence and robust evidence for the effectiveness of CBEs has not been published. Wide-spread adoption of chair-based exercise should only be contemplated if it is shown to be both clinical and cost effective. Collating what is already known about CBE programmes for frail older people will guide current practice and identify areas for further development and research. 

## 2. Objective

To examine the beneficial and harmful effects of exercise programmes performed primarily in the seated position (CBE) for frail older people who are unable to perform standard evidence-based exercise programmes.

## 3. Methods

We performed a systematic review of existing literature with the Cochrane Collaboration recommended standardised search strategy and used the PRISMA (Preferred Reporting Items for Systematic reviews and Meta-Analyses) methodology for reporting [7, 8].

### 3.1. Eligibility Criteria

Studies were considered to be eligible for inclusion using the following criteria:

- study type: randomised controlled trials and other controlled trials,
- population: participants all 65 years and over or where the mean and standard deviations indicated that the majority of participants were 65 years and over. Participants who were described as or implied to be frail were eligible for inclusion. This broad based definition of frailty was used to ensure that suitable papers were not omitted,
- intervention: exercise programmes performed primarily in the seated position. Studies which described “high intensity” interventions or used complex equipment (e.g., treadmills and multigym) were excluded due to clinical relevance,
- outcomes: all outcomes were included as we believed that a wide range of outcome measures would be employed and we wanted to capture all beneficial and harmful effects,
- setting: all settings included.

### 3.2. Data Sources

An electronic search was conducted using the following databases: Medline, CINHAL, Psychinfo, Bandolier, Cochrane, DARE, Health Technology Assessment (HTA) reports, NHS Economic Evaluation Database, and AMED. Secondary references were also accessed and wherever necessary and possible, personal communications with researchers were undertaken. The Profane (Prevention of Falls Network Europe (http://www.profane.eu.org/)) website was also searched. Suitable trials published between 1990 and February 2011 were eligible for consideration. The older person as a population has changed in recent years and continues to do so, and therefore only papers from 1990 onwards were considered in order to represent the contemporary population.

### 3.3. Search Strategy

The search terms and strategy used to identify the papers are outlined. The same search terms were used in all of the electronic databases identified restricting articles from 1990 to February 2011.

### 3.4. Study Selection

All studies identified in the search were screened for eligibility by a primary reviewer, with duplicates and ineligible studies removed. Full text versions of the remaining studies were then further assessed for eligibility by the primary reviewer. A second reviewer independently assessed eligibility of the studies classified as suitable for inclusion. Where there was disagreement a third reviewer adjudicated. Of the studies deemed not suitable for inclusion, a 10% audit was performed by the second reviewer.

*Summary of Search Strategy (Search Terms). *Rehabilitation, Benefits, Human, Falls; older person; Frail; frailty; Frail OR older AND chair AND exercises; frail* AND older*; frailty AND exercise; moderate exercise; chair based exercise; chair based exercise AND older person; light exercise; exercise AND older person; chair based exercise older person; chair based rehabilitation benefits; moderate exercises AND older person; light exercises AND older person; frail OR older person AND exercises; frail AND older person; chair AND exercise AND older person; aging AND mental AND health AND exercise.

### 3.5. Data Extraction and Synthesis

Due to diversity of outcome measures, meta-analyses of studies were not feasible. Relevant findings, interpretation, and implications for clinical practice and research are therefore presented in narrative form. Findings and comments are presented on the following key areas: effects of CBE, outcomes and evaluation of CBE, quality of the evidence, and implications for practice and future research.

### 3.6. Quality Appraisal

Quality assessment of studies was conducted using RevMan version 5 [7] and the Jadad Scale was used [9]. The Jadad scale was chosen due to the holistic nature of the measure and its applicability to evaluating complex interventions for frail older people. This scale includes three items directly related to systematic bias reduction: randomisation, blinding, and description of withdrawal and dropouts. The tool allows for a range of 0–5 points and studies having been awarded ≤2 points are considered to be of poor quality (high risk of bias).

## 4. Results

**Figure 1:**
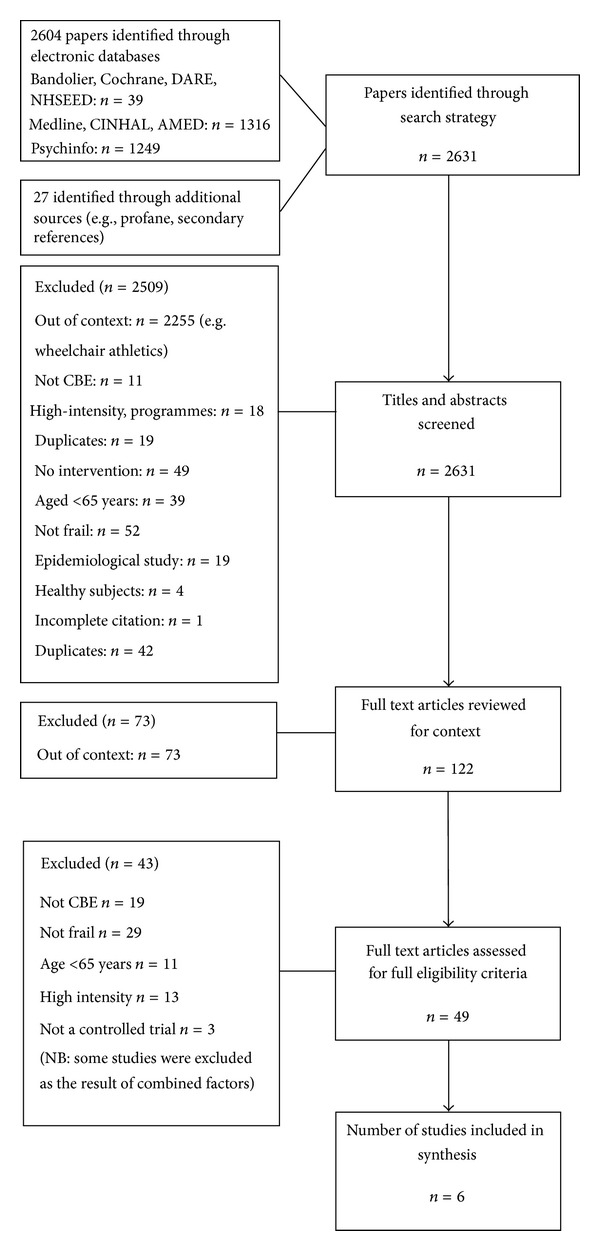
Study selection and results.

The initial search yielded 2631 abstracts, from which 49 articles were read in full, identifying six studies for inclusion. The results of the searches and screening for eligibility are presented in Figure 1.

A summary of the characteristics of the eligible studies is provided in Table 1 and key points are presented below.

### 4.1. Participants and Setting

All six studies were small (range of participants from 20 to 82) and at single sites. Participants ranged from 70 to 99 years of age. Three of the studies were completed in care homes [10–12], one in a day centre [13], one in a geriatric hospital [14], and one in a community setting [15]. Participants studied included long-term care residents [10–12], post-hip-fracture patients [14], those diagnosed with heart failure [15], and patients with cognitive impairment [12].

### 4.2. Intervention

Each study evaluated a different form of CBE intervention, with variations in format, delivery, frequency, and intensity. Interventions ranged in duration from 6 weeks [13] to 6 months [10] with frequency of exercise sessions ranging from daily [12] to three times a week [10, 11, 13]. The duration of each session also varied with one study reporting 20 minutes per session [14] and two others reporting up to 60 minutes per session [10, 11].

### 4.3. Effects of CBE Programmes

A total of 26 outcome measures (range 3–9) were used in the included studies. For the purposes of this systematic review, we divided them into three groups: mobility and function [10, 11, 13–15], mental health [10, 12, 14, 15], and cardiorespiratory fitness [11, 14, 15].

No adverse effects were reported suggesting that CBE programmes appear to be safe and appropriate for this population.

#### 4.3.1. Mobility and Function

Three of the studies examined mobility and function, using a variety of outcome measures related to function, gait, and balance. 

Two studies showed improvements in timed up and go (TUG) scores [10, 13]. One study [10] noted an eighteen-second decrease in TUG scores between the intervention and control group.

Functional measures such as sitting to standing were used in two studies [11, 13]. A 66% improvement in the 30-second chair stand for the exercise group was reported by Hruda et al. [11]. Thomas and Hageman [13] reported that through twice weekly exercise adherence; sit to stand time improved by 22% before and after intervention. 

Gait speed, stability, and distance were used as physical outcome measures in several studies [11, 13, 15]. In day centre participants [13] fast gait time decreased by 4.1% (*P* = .06) as did the number of steps (0.8%; *P* not stated) and the number of steps during normal gait (5.2%; *P* not stated). Witham et al. [15] reported no significant differences between groups using the 6-minute walk distance at three and six months. Significant changes in daily activity measured using accelerometry were however reported at the 6-month point.

Muscle strength was used by three studies to determine the exercise benefits of interventions [10, 13, 14]. Two studies reported significant changes in muscle strength between groups and postintervention [11, 13]. One study [14] reported improved grip strength in both the control and intervention group with no significant difference between the groups. 

In the other two studies significant improvements were identified in eccentric (44%) and concentric (60%) average power (*P* = 0.05) [11], and improvements in grip strength (10%, *P* = 0.04) were reported [13]. One study [13] reported a decline in iliopsoas (3.6%) and dorsiflexor strength (8.0%), both of which are necessary to maintain hip and ankle flexion and thus optimal gait patterns with a reduced risk of falls. Hruda et al. [11] noted an improvement in 8 foot up and go with a negative correlation between 8 foot up and go and concentric power. This suggests an increased cadence but reduced postural stability during gait.

Witham et al. [15] reported that the Functional Limitations Profile (UK adaptation of Sickness Impact Profile) suggested a preservation of functional capacity. It is noted however that this questionnaire was administered during a home visit which may have resulted in bias as the result of the home setting. 

Fear of falling was examined by Nicholson et al. [14] using the Falls Efficacy Scale with levels of change not reported. Falls risk and falls rate were not examined by any other studies. 

#### 4.3.2. Mental Health

Four studies examined mental health with no negative changes reported in any of the studies [10, 12, 14, 15]. The Mini Mental State Examination (MMSE) was utilised in two of the studies [10, 12] and significant improvements in cognition were demonstrated in both studies. 

Van de Winckel et al. [12] also reported improvements though a post hoc contrast at 6 weeks and 12 weeks. MMSE was not assessed after completion of the intervention, and therefore no inference can be made over long-term cognitive benefits. No significant changes were noted in behaviour as measured by the Beoordlingschaal voor Oudere Patienten (BOP) (evaluation scale for elderly patients).

The Hospital Anxiety and Depression Scale was used in one study [15] which demonstrated reduced levels of depression at three and six months. Depression was measured using the Beck Depression Inventory in one study [14] with reduced levels of depression reported in both the control and exercise group and no statistically significant differences between the groups noted. 

#### 4.3.3. Cardiorespiratory

Three studies examined effects on cardiorespiratory fitness [11, 14, 15]. Witham et al. [15] reported nonstatistically significant changes in the exercise in terms of the 6-minute walking distance (2.1% change at 3 months *P* = 0.23 and 4.4% change at 6 months, *P* = 0.84).

Significant improvements in systolic blood pressure and heart rate in postsurgical participants were reported by Nicholson et al. [14].

### 4.4. Quality Appraisal

The Jadad scale revealed a quality assessment summary score which found the quality to be poor in all six studies. All of these 6 studies were small (range 20–82 participants) and single centred. A summary of the quality appraisal of all studies can be found in Table 2.

## 5. Discussion

To our knowledge this is the first systematic review of chair-based exercise programmes specifically in a frail elderly population identifying the need for this work. Although the body of literature surrounding CBE is broad and diverse (e.g., wheelchair athletics and spinal injuries), literature specifically for frail older people appears sparse. This systematic review only found six studies examining the effects of chair-based exercises provided specifically for frail older people. All studies were small (range of participants 20–82) and performed in single sites. The duration of the interventions varied (range 6 weeks–6 months) as did the age (range 70 years–99 years). Meta-analysis was not feasible due to the diversity of the outcome measures used. In addition, the disparity in interventions and settings made comparison between studies challenging. Conflicting poor quality evidence regarding the effectiveness of CBE programmes provides little guidance for clinicians, care providers, and commissioners.

Conducting research and particularly randomised controlled trials in frail elderly populations is often more challenging and complex in comparison to younger healthy adults [16]. The diversity of the older person in terms of culture, health beliefs, age, and functional abilities makes it more difficult to recruit truly representative study populations which in turn can impact on the study findings. This is apparent where Hruda et al. [11] make note of a trend for subjects in the control group to be younger than those in the intervention group potentially confounding comparisons. The effect of the intervention may also be influenced by the heterogeneity of older people as participants of studies identified by Nicholson et al. [14] who suggest that “the effect of the exercise intervention may have been obscured by the large differences between individuals.” 

Based on this review defining chair-based exercise as an intervention for frail older people would appear challenging. All studies in this review described different interventions delivered in different setting and with a very different focus. These disparities identify the flexibility of CBE to adapt to specific needs and contexts. However, the lack of standardisation limits the ability of this review to clearly define CBE programmes for frail older people and determine their effectiveness. The diversity of programmes in terms of duration, frequency, exercise type and intensity, and followup clearly identifies a lack of consensus on the fundamental principles of chair-based exercise programmes for frail elderly populations. 

This review has identified variations in interventions in key areas such as target population, length, and frequency and setting which need to be carefully considered and related to programme aims. For example the length of an intervention needs to be carefully considered to ensure that it maximises change; both Nicholson and Thomas report that their intervention was too short and at suboptimal frequencies to demonstrate changes. 

All studies noted high adherence rates. Motivational reasons may have contributed to this in some studies; for example, participants in the post-hip-fracture study may have had a strong desire to return home [14].

A total of 26 diverse outcome measures were found within the included studies (range 3–9) acknowledging the wide-ranging perceived effects of CBE programmes. Benefits in the domains of mobility and postural stability, cardiorespiratory fitness, and mental health were identified. However findings and the strength of findings were contradictory between studies. The included studies in this review provide encouragement for the use of CBE for frail older people with significant improvements in function, mobility, and mental health reported. It is important to take note of these encouraging findings in a vulnerable frail elderly population where guidance over appropriate physical activity is lacking. This review has identified that chair-based exercise programmes have the potential to provide a safe and accessible form of exercise for a vulnerable population who cannot participate safely in other forms of exercise. 

 The purpose and role of CBE programmes however need to be established to ensure appropriate evaluation. Careful selection of outcome measures underpinned by the focus and rationale for the intervention is imperative for accurate evaluation and to ensure that treatment effects are not missed. 

This review has some limitations. The findings are limited due to the relatively small number and poor quality of the studies identified making it difficult to form clear conclusions regarding the effects of CBE programmes. The challenge of defining chair-based exercise programmes has been highlighted within the review and as such it is possible that relevant literature was not included in the review due to the methodology of the search strategy. Searching for literature of this kind is challenging with few studies explicitly stating chair-based or -seated exercise programmes within key terms and titles. A further limitation of this review is that the studies were selected using a broad description of frailty. There are frailty definitions in the literature [17, 18], but they have narrow criteria and very few studies have used such specific definitions. We deliberately used wider inclusion criteria for frailty so that we captured all available literature.

## 6. Conclusions

The quality of the evidence base for CBEs is low, as a result of small single sited studies, the use of varied outcome measures, and flawed methodological techniques. Whilst benefits are noted within the included studies, methodological issues reduce confidence in the findings. However, there is sufficient evidence to suggest that benefit is plausible. A consensus process as to the purpose and definition of CBEs followed by large well-designed randomised controlled trials to test the effectiveness and cost-effectiveness of CBE is justified.

## Figures and Tables

**Table 1:** Characteristics of the selected studies.

Author	Study type	Sample size	Setting	Focus	Intervention	Adherence	Outcome measures	Main/significant findings
Baum et al. (2003) [10]	Prospective, randomised, controlled, semicrossover trial	20	Nursing home/assisted living scheme (USA)	Upper/lower limb strength and flexibility related to function	Seated ROM and progressive resistance, 1 hour three times per week; 6 months	Mean attendance/adherence 80%	Timed Up and Go Berg Balance Scale Physical Performance Test (PPT) Mini Mental State Examination (MMSE)	Positive intervention effect for all outcomes as below: TUG—18 seconds faster, effect size = 0.54 Berg Balance—4.8 better, effect size = 0.32 PPT—1.3 better, effect size = 0.40 MMSE—3.1 better, effect size = 0.54 NB: Effect sizes are the difference between intervention and control in standard deviation units

Hruda et al. (2003) [11]	Randomised controlled trial	25	Long-term care facility (Canada)	Lower limb strength related to function	Progressive lower body resistance exercises; ≤1 hour three times per week; 10 weeks	Mean attendance/adherence 71%	Eight foot up and go 30 second chair stand 6 meter walk 30 second chair stand Eccentric/concentric average power	Significant improvement in eight foot up and go speed (*P* = 0.05) Significant improvement in 30 second chair stand (*P* = 0.05) Negative correlation between eight foot up and go and concentric power (*P* = 0.05)

Van de Winckel et al. (2004) [12]	Randomised controlled trial	25	Dementia registered Residential home (Belgium)	Mood and cognition in presence of dementia	Music supported chair movement exercise (30 minutes daily) for 3 months (resistance not included)	Mean attendance/adherencenot reported	Mini-Mental State Examination Beoordelingsschaal voor Oudere Patienten (BOP)	Improvement in MMSE in exercise group before and after intervention (mean 12.87 versus 15.53, effect size = 0.5, *P* = 0.001) No significant differences in behaviour (no items in the BOP scale identified significant improvement in exercise group)

Thomas and Hageman (2003) [13]	Before and after test	28	Day centre (USA)	Lower limb strength and function in presence of dementia	Moderate intensity progressive resistance training of hip extensors, flexors, and dorsiflexors with Theraband for up to 3 days per week for six weeks	Mean attendance/adherence 63%	Bilateral muscle testing Walking speed Sit-stand x5 Standing balance Timed Up and Go (TUG) Body Mass Index Mini-Mental State Examination Gait Assessment Rating Scale	Of participants who exercise at least twice a week the following was noted: 22% improvement in sit-stand time 14% improvement in TUG 10.1% improvement in average grip strength 15.6% in average quadriceps strength 9.9% in usual gait time 5.4% in fast gait time

Nicholson et al. (1997) [14]	Quasi experimental, nonrandomised control group before and after test	71	Multidisciplinary team geriatric hospital (South Africa)	After-hip-fracture rehabilitation	Seated “High paced” “choreographed” “complex movements.” 60% max heart rate for 20 minutes	Mean attendance/adherence 92%	Falls Efficacy Scale Habitual Physical Activity Questionnaire Beck Depression Inventory (BDI) Mini Mental State Examination	Increased levels of grip, mood and confidence before and after in both exercise and control group. Between group (control and exercise) differences nonsignificant: Grip Strength, *P* = 0.29 FES Confidence, *P* = 0.99 FES Fear, *P* = 0.72 BDI, *P* = 0.80 Improved systolic blood pressure and heart rate (*P* < 0.01) over exercise and recovery period

Witham et al. (2005) [15]	Randomised single blind controlled trial	82	Community (UK)	Exercise capacity, function, and health status in presence of chronic heart failure	3 month supervised resistance exercise classes (unclear type of resistance) twice per week followed by home exercises with the aid of video/audio cassette (no face to face). Control was standard care	Mean attendance/adherence 83%	Six-minute walk Accelerometry Guyatt (chronic heart failure questionnaire) Hospital Anxiety and Depression Scale Philadelphia Geriatric Morale Scale	Nonsignificant improvement in mental health (HADS, *P* = 0.10) Nonsignificant improvement in walking distance (6-minute walk, *P* = 0.84)

**Table 2:** Summary of quality appraisal.

Author	Adequate sequence generation?	Allocation concealed?	Blinding appropriate?	Incomplete outcome data addressed?	Free of selective reporting?	Free of other bias?	Jadad score
Baum et al. (2003) [10]	Yes (computer-generated algorithm)	Unclear (assignment by opening sealed envelopes supplied in sequence by the study coordinator)	Yes (all tests administered by blinded therapists. MMSE administered by two blinded medical students and research nurses)	Unclear (13%) after baseline data missing (death/acute illness) One death during follow-up period	Unclear (details of nursing home patients provided but not the assisted living patients)	No (selection bias suggested in text)	0

Hruda et al. (2003) [11]	Unclear (no methodology discussion within the text)	Yes (assigned in a lottery format *≈*1 : 2 ratio (control *n* = 10; intervention *n* = 20))	Unclear (no methodology discussion within the text)	Unclear (no method discussion within the text)	Unclear (no method discussion within the text)	Unclear (no method discussion within the text)	2

Van de Winckel et al. (2004) [12]	No (no sequence generation evident)	No (randomisation performed by tossing a coin. It is unclear who or how many times the coin was tossed)	No (physical tests performed by Physio who performed the exercise groups)	Yes (only one dropout—hip fracture)	Yes (no selective reporting evident)	Yes	0

Thomas and Hageman (2003) [13]	No (no randomisation)	No (no randomisation/blinding	No (no blinding)	Unclear (trial in place for 6 weeks and authors report “only 1/3 of subjects completing less than 11 sessions.” It is also not clear what happened—were they discontinued?)	Yes (no selective reporting apparent)	Yes	2

Nicholson et al. (1997) [14]	No (randomisation and assignment reported to be “not possible”)	Yes (randomisation not possible; research assistant blinded to allocation)	No (it is not clear who and how subjects were allocated)	Unclear (unclear completion or drop-out levels and “some missing data” are also noted)	Unclear (no selective reporting apparent; however, data might be incomplete)	Yes (no other bias apparent)	2

Witham et al. (2005) [15]	Yes (computer-generated numbers)	No (primary researcher wrote cards with “Exercise” or “Control” out. The cards were then placed into numbered envelopes (1 upwards) which were opened in sequence as each new patient was randomised)	No (primary researcher was able to identify sequence of allocation)	Yes (82% attendance rate—primary author contacted and provided details of absences)	Yes (no selective reporting evident)	Yes	0
